# Multimodal therapy for primary ureteral small cell neuroendocrine carcinoma with high-grade urothelial component: case report and literature review

**DOI:** 10.3389/fruro.2025.1615270

**Published:** 2025-11-03

**Authors:** Yaqi Li, Yugang Deng, Siwei Ma, Jie Liu

**Affiliations:** ^1^ Henan University People’s Hospital, Zhengzhou, Henan, China; ^2^ People’s Hospital of Henan Medical University, Zhengzhou, Henan, China; ^3^ Henan Provincial People’s Hospital, Zhengzhou, Henan, China

**Keywords:** neuroendocrine tumors, ureteral neoplasms, immune checkpoint inhibitors, neoadjuvant chemotherapy, combined modality therapy

## Abstract

Primary small cell neuroendocrine carcinoma (SCNEC) of the ureter is an exceptionally rare and aggressive malignancy, characterized by rapid progression and poor prognosis. Evidence regarding treatment with immune checkpoint inhibitors (ICIs) combined with neoadjuvant chemotherapy remains limited. We report the first documented case of ureteral SCNEC treated with a multimodal strategy incorporating programmed death-ligand 1 (PD-L1) inhibitors. A 57-year-old woman presented with a 15-day history of gross hematuria and 4 days of progressive left flank pain. Imaging revealed a left distal ureteral mass with hydronephrosis and suspected iliac lymphadenopathy. Stage IV high-grade urothelial carcinoma with small cell neuroendocrine differentiation of the ureter with regional lymph node metastases, confirmed by histopathology and immunohistochemistry (synaptophysin+, CD56+, Ki67 >75%). The patient received four cycles of neoadjuvant etoposide plus carboplatin chemotherapy, followed by radical left nephroureterectomy with bladder cuff excision. Adjuvant therapy included intravesical pirarubicin and six cycles of dual PD-1 blockade with toripalimab and vedicitumab. Post-neoadjuvant imaging showed a 60% reduction in tumor size. Pathology revealed R0 resection with marked tumor regression (Ki67 reduced to 40%). No recurrence was observed at 12-month follow-up. This case demonstrates the potential efficacy of integrating ICIs with standard treatment for advanced ureteral SCNEC. The durable response observed underscores the need for further research into early immunotherapy use and biomarker-guided therapeutic strategies.

## Introduction

Small cell neuroendocrine carcinoma (SCNEC), most commonly arising in the lungs and gastrointestinal tract, accounts for fewer than 0.05% of all genitourinary malignancies. Since its initial description in 1986, fewer than 80 cases of primary ureteral SCNEC have been reported. This rare neoplasm is characterized by high mitotic activity, early metastasis, and poor prognosis, with a median survival of 12–24 months despite aggressive treatment. Although radical surgery remains the cornerstone of management, the local recurrence rate exceeds 60%, largely due to undetected micrometastases at diagnosis. Recent evidence from small cell lung cancer (SCLC) trials suggests that platinum-based chemotherapy combined with immune checkpoint inhibitors (ICIs) may improve outcomes. However, the application of this strategy to ureteral SCNEC has not been previously reported. Herein, we describe the first case of advanced ureteral SCNEC successfully managed with a multimodal regimen involving neoadjuvant chemotherapy, radical surgery, and adjuvant PD-1 blockade.

## Case presentation

A 57-year-old non-smoking woman presented with painless gross hematuria lasting 15 days and progressive left flank discomfort for 4 days. Her medical history was unremarkable. Physical examination revealed left costovertebral angle tenderness, without palpable masses. Urinalysis showed significant microscopic hematuria (RBC >100/HPF) and pyuria (WBC 20–30/HPF).

**Figure 1:**
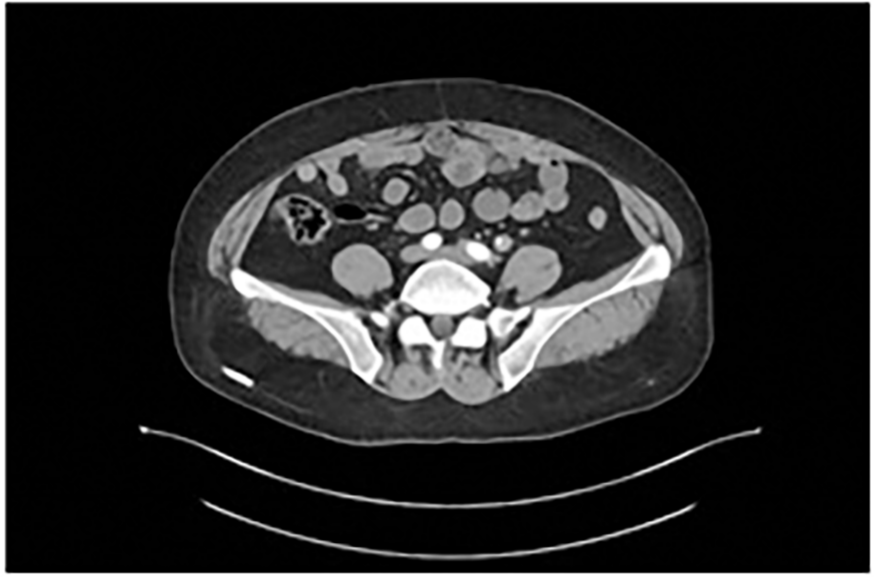
Contrast-enhanced abdominopelvic CT demonstrates circumferential thickening of the distal left ureter (1.8×1.5 cm), associated with proximal hydroureter.

**Figure 2:**
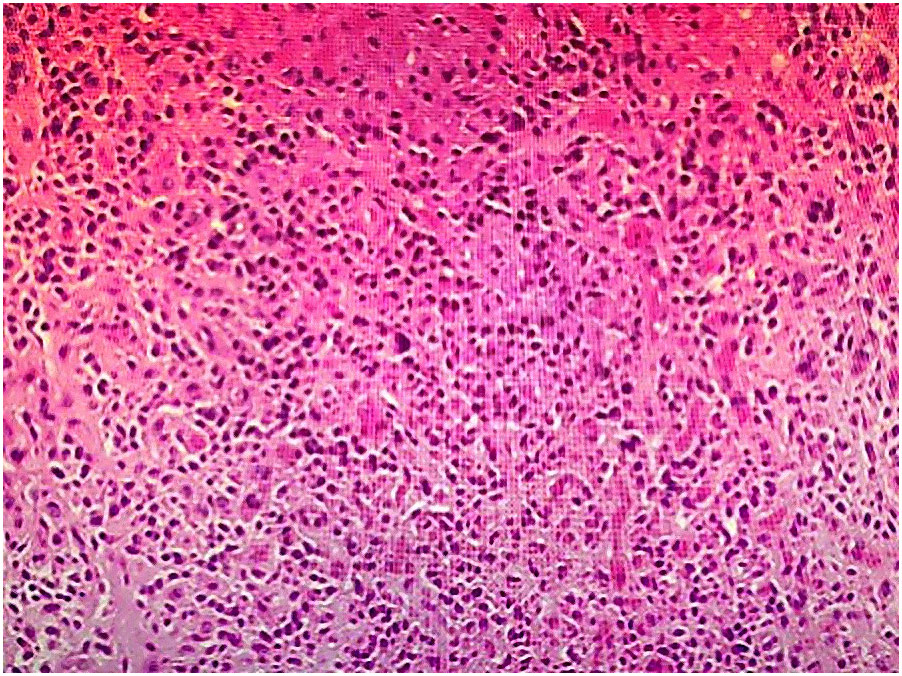
Histological section of the resected ureter (Hematoxylin and eosin staining, ×200), showing residual small cell neuroendocrine carcinoma with muscularis propria infiltration.

Contrast-enhanced abdominopelvic computed tomography (CT) revealed concentric thickening of the distal left ureter (1.8 × 1.5 cm), with upstream hydroureteronephrosis and an enlarged ipsilateral external iliac lymph node (short axis: 1.2 cm) (**Figure 1**). ^18F-FDG PET/CT demonstrated intense radiotracer uptake in the ureteral lesion (SUVmax 8.2) and in the lymph node (SUVmax 5.6). Ureteroscopic biopsy revealed sheets of small hyperchromatic cells with nuclear molding and a high mitotic index (>20 mitoses/10 HPF) (**Figure 2**).

**Figure 3:**
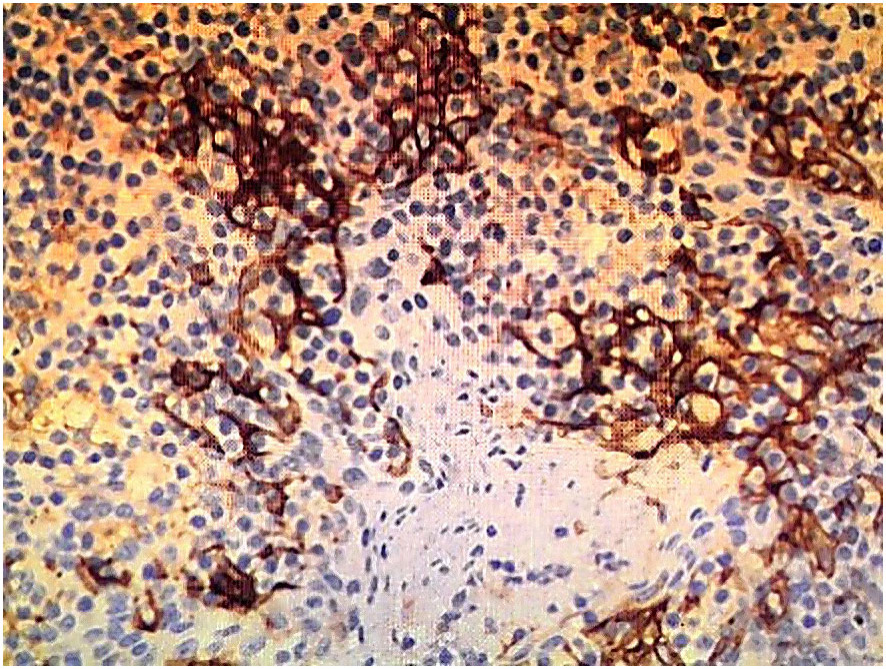
PD-L1 immunohistochemical staining using the EnVision method (×200), showing low expression (CPS = 2).

Immunohistochemical staining confirmed the diagnosis of SCNEC: synaptophysin (diffuse+), CD56 (membranous+), chromogranin A (focal+), and a Ki67 proliferation index exceeding 75%. PD-L1 expression, assessed using the 22C3 pharmDx assay, was low (combined positive score [CPS] = 2) (**Figure 3**). Staging investigations excluded metastatic disease outside the regional lymph nodes.

**Figure 4:**
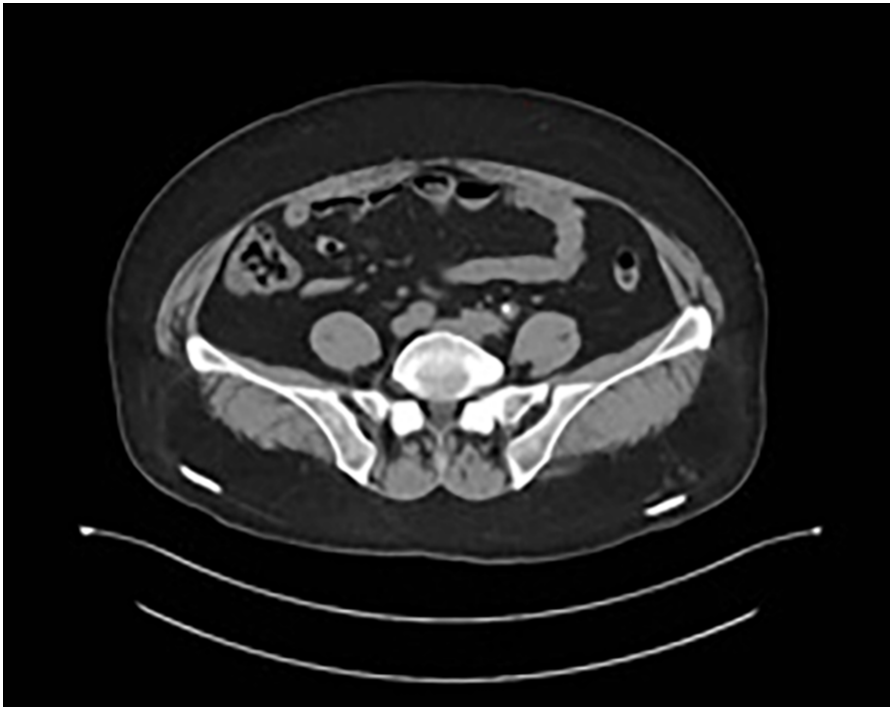
Follow-up CT after neoadjuvant chemotherapy demonstrating marked tumor regression (residual lesion: 0.7 × 0.6 cm) and normalization of ureteral caliber.

The patient underwent four cycles of neoadjuvant chemotherapy with etoposide (100 mg/m² on days 1–3) and carboplatin (AUC 5 on day 1), administered every 21 days. Follow-up CT imaging demonstrated substantial tumor regression, with a residual lesion measuring 0.7 × 0.6 cm and normalization of ureteral caliber (**Figure 4**).

Subsequently, she underwent radical left nephroureterectomy with bladder cuff excision and regional lymphadenectomy. Intraoperatively, the tumor was confined to the ureteral wall, with no evidence of serosal invasion.

Histopathology revealed residual high-grade urothelial carcinoma (UC) with small cell neuroendocrine differentiation (SCNEC), with the neuroendocrine component estimated to comprise approximately 70% of the tumor area (semi-quantitative visual estimate of approximately 70%, pending confirmatory review by a pathologist). All surgical margins were negative, and none of the 12 resected lymph nodes harbored metastasis. Post-treatment Ki67 index had decreased to 40%.

Adjuvant therapy included six cycles of toripalimab (240 mg) and vedicitumab (120 mg) administered every 3 weeks, along with monthly intravesical instillations of pirarubicin (50 mg × 6). Surveillance cystoscopy and quarterly CT scans over 12 months revealed no evidence of recurrence.

## Discussion

Small cell neuroendocrine carcinoma (SCNEC) is a high-grade neuroendocrine tumor most commonly originating in the lungs and gastrointestinal tract. Its occurrence in the urinary system is exceedingly rare, accounting for just 0.05% of all neuroendocrine carcinomas, with the bladder being the most frequent genitourinary site. Ureteral SCNEC is particularly uncommon, with fewer than 80 cases reported since it was first described by Ordonez et al. in 1986 (1).

Patients with ureteral SCNEC typically present with flank pain and hematuria—flank pain usually results from hydronephrosis due to ureteral obstruction, while hematuria is often attributable to tumor invasion of the urothelial vasculature. Paraneoplastic syndromes are infrequently observed. Although the exact pathogenesis remains unclear, smoking is considered a significant risk factor, with over half of patients having a smoking history. Two main hypotheses exist regarding tumor origin:

1. Neuroendocrine Cell Origin Hypothesis: Suggests derivation from neural crest–derived enterochromaffin cells that migrate during embryogenesis.
2. Pluripotent Epithelial Cell Hypothesis: Proposes that the tumor arises from multipotent urothelial cells capable of divergent differentiation, potentially explaining the frequent coexistence of other histologic subtypes, such as urothelial, squamous, glandular, and sarcomatoid components (2).

Molecular studies have identified frequent TP53 mutations, as well as chromosomal deletions (10q, 4q, 5q, 13q) and gains (8p, 5p, 6p, 13p), implicating genetic instability in ureteral. Imaging modalities, including CT and MRI, may reveal ureteral wall thickening, luminal narrowing, or periureteral invasion. However, these findings lack specificity and often overlap with other ureteral malignancies. Therefore, histopathological evaluation remains the gold standard for diagnosis. Immunohistochemistry (IHC) plays a crucial role, with positive staining for neuroendocrine markers such as synaptophysin (SYN), chromogranin A (CgA), neuron-specific enolase (NSE), and CD56 aiding diagnosis. Although CD56 lacks specificity, its absence essentially excludes the diagnosis. Our patient exhibited SYN+, CD56+, NSE+, and CgA− staining, consistent with SCNEC (3).

Due to the rarity of this tumor, there is no established treatment guideline. Nonetheless, evidence suggests that ureteral SCNEC differs biologically from conventional urothelial carcinoma and warrants distinct therapeutic strategies (4). Multimodal treatment approaches—comprising surgery, radiotherapy, and systemic chemotherapy—are widely advocated. Radical nephroureterectomy with bladder cuff excision is the standard surgical procedure for localized disease (5). However, prognosis remains dismal, with systemic relapse being common. Zhong et al. reported regional recurrence in 9.4% and distant metastases in 25% of cases within a short follow-up period (4). Similarly, Ping et al. described peritoneal relapse 4 months post-radical surgery in a pT2N0M0 patient (6)., and Wang et al. observed rapid dissemination with a 12-month survival in a pT3N0M0 case (7). These findings underscore the importance of systemic therapy, even in apparently localized disease.

Neoadjuvant chemotherapy (NAC), particularly when combined with immune checkpoint inhibitors (ICIs), is emerging as a promising approach. NAC can reduce tumor burden, increase the likelihood of complete resection, and address micrometastases. Ahsaini et al. reported a case of ureteral SCNEC with a 3-year recurrence-free survival following NAC (8). In our case, the tumor showed excellent responsiveness to etoposide–carboplatin, confirming platinum sensitivity. Farooq et al. also documented complete pathological response after neoadjuvant cisplatin–etoposide chemotherapy (9).While randomized trials are lacking, accumulating case-level evidence supports the value of NAC in managing SCNEC.

The landscape of SCNEC treatment is evolving with the integration of immunotherapy. In extensive-stage small cell lung cancer (SCLC), chemoimmunotherapy has become the standard of care. A recent meta-analysis validated the efficacy and safety of PD-L1 inhibitors in this setting (10). Given the high recurrence rate and lack of standardized treatment in ureteral SCNEC, we employed an innovative regimen combining neoadjuvant chemotherapy with postoperative dual PD-1 blockade (toripalimab and vedicitumab). This approach resulted in a 60% reduction in tumor volume, with pathology confirming R0 resection, minimal residual disease (30% viability), and a Ki67 index decline from >75% to 40%. The patient remained disease-free at 12 months, demonstrating the potential of this multimodal approach to delay progression and reduce metastasis risk, especially in tumors with high-grade urothelial carcinoma components.

In the present case, histopathology confirmed a mixed tumor composed of high-grade urothelial carcinoma (UC) and small cell neuroendocrine carcinoma (SCNEC). While SCNEC is known for its aggressive course and often limited response duration to immune checkpoint inhibitors (ICIs), the coexisting UC component warrants additional consideration, as substantial clinical evidence supports the sensitivity of UC to PD-1/PD-L1–targeted therapies.

Multiple phase III trials have demonstrated the benefit of ICIs in advanced UC. In the KEYNOTE-045 study, pembrolizumab significantly improved overall survival (OS) in platinum-refractory metastatic UC compared with chemotherapy (median OS 10.3 vs. 7.4 months) and achieved higher objective response rates with fewer high-grade adverse events (11). The JAVELIN Bladder 100 trial established switch-maintenance avelumab as a standard of care, prolonging OS from 14.3 to 21.4 months in patients without progression after first-line platinum-based chemotherapy (11). First-line pembrolizumab has also shown durable responses in cisplatin-ineligible, PD-L1–positive UC patients (KEYNOTE-052) (12).

Regarding upper tract urothelial carcinoma (UTUC), which includes ureteral primaries, subgroup analyses suggest some variability in ICI responsiveness. While second-line response rates may be modestly lower in UTUC compared with bladder UC (e.g., JAVELIN Bladder 100 subgroup: ORR ~11% vs. 18%) (12), first-line immunotherapy trials (KEYNOTE-052, IMvigor210) have reported comparable ORRs between UTUC and bladder UC (12). This indicates that the UC component in mixed histologies, such as in the present case, could contribute meaningfully to observed ICI efficacy.

Therefore, the durable disease control in our patient may not be solely attributable to the SCNEC component’s response but also to the established PD-L1–oriented therapy sensitivity of the UC component. This aligns with therapeutic strategies in UC where platinum-based chemotherapy followed by ICI maintenance or consolidation is increasingly recognized as an effective sequence (13).

Radiotherapy may serve as an alternative or adjuvant modality in selected high-risk or locally advanced cases. Farooq et al. described a patient who achieved recurrence-free survival for 12 months following NAC and adjuvant pelvic chemoradiotherapy (50 Gy) (9). However, caution is warranted when combining radiotherapy with immunotherapy. Qing et al. reported hyperprogression in a patient treated with PD-L1 inhibitors plus radiotherapy, emphasizing the need for vigilant monitoring and patient selection (1). In summary, our experience highlights the promising role of multimodal therapy—including NAC, radical surgery, and adjuvant ICIs—in managing ureteral SCNEC. Future research should focus on personalized treatment strategies guided by molecular profiling and biomarker assessment.

## Conclusion

Primary ureteral small cell neuroendocrine carcinoma (SCNEC) is an exceedingly rare and aggressive malignancy with limited treatment guidelines and poor prognosis. This report adds to the scarce literature and highlights the potential benefit of integrating neoadjuvant chemotherapy with immune checkpoint inhibitors (ICIs) in a multimodal treatment strategy. Our patient achieved significant tumor regression, pathological response, and sustained disease-free survival following this approach.

Given the presence of a substantial high-grade urothelial carcinoma component with small cell neuroendocrine differentiation, interpretation of immunotherapy benefit should incorporate evidence from UC populations, where PD-1/PD-L1 blockade has demonstrated proven survival benefit (13).

Future research should focus on expanding therapeutic options through clinical trials, incorporating molecular profiling, and identifying predictive biomarkers to guide individualized treatment. Potential molecular targets—such as C-kit, EGFR, BCL2, and CD56—identified in prostatic small cell carcinoma warrant further investigation in ureteral SCNEC. Additionally, dynamic treatment monitoring and long-term outcome data are essential to optimize clinical decision-making in this challenging disease.

## Data Availability

The original contributions presented in the study are included in the article/supplementary material. Further inquiries can be directed to the corresponding author.

## References

[1] Qing D Peng L Cen F Huang X Wei Q Lu H . Hyperprogression after immunotherapy for primary small cell neuroendocrine carcinoma of the ureter: a case report. Front Oncol. (2021) 11:696422. doi: 10.3389/fonc.2021.696422, PMID: 34485132 PMC8416087

[2] Farci F Manassero F Baldesi R Bartolucci A Boldrini L Selli C . Primary small cell carcinoma of the ureter: case report and review of the literature. Med (Baltimore). (2018) 97:e11113. doi: 10.1097/MD.0000000000011113, PMID: 29901633 PMC6023684

[3] Wang C Liu S Zhang Y Feng F Qiu Z . A case report of primary small cell neuroendocrine carcinoma of the ureter. J Clin Surg. (2022) 30:717–8. doi: 10.3969/j.issn.1005-6483.2022.08.005

[4] Zhong W Lin R Zhang L Jin C Li X He Q . Clinicopathologic characteristics, therapy, and outcomes of patients with primary ureteral small cell carcinoma: a case series and systematic review. Onco Targets Ther. (2017) 10:4105–11. doi: 10.2147/OTT.S138769, PMID: 28860819 PMC5566501

[5] Maruyama Y Araki M Wada K Yoshinaga K Mitsui Y Sadahira T . Long-term ureteroscopic management of upper tract urothelial carcinoma: 28-year single-centre experience. Jpn J Clin Oncol. (2021) 51:130–7. doi: 10.1093/jjco/hyaa132, PMID: 32715306

[6] Ping JH Chen ZX Jiong Q Han YQ Nong X . Small cell neuroendocrine carcinoma of the ureter: a case report and literature review. Oncol Lett. (2014) 7:728–30. doi: 10.3892/ol.2013.1757, PMID: 24520292 PMC3919792

[7] Wang W Liu G Li Y Siriwardane U Ma H . Neuroendocrine carcinoma of the ureter: a case report and literature review. Oncol Lett. (2016) 11:257–60. doi: 10.3892/ol.2015.3899, PMID: 26870199 PMC4727205

[8] Ahsaini M Riyach O Tazi MF El Fassi MJ Farih MH Amarti A . Small cell neuroendocrine carcinoma of the urinary tract successfully managed with neoadjuvant chemotherapy. Case Rep Urol. (2013) 2013:598325. doi: 10.1155/2013/598325, PMID: 24024065 PMC3759270

[9] Farooq M Daniel S Joel A Thampi JN . Successful management of small cell neuroendocrine carcinoma of the ureter with neoadjuvant chemotherapy and adjuvant chemoradiation: case report and literature review. BMJ Case Rep. (2021) 14:e240613. doi: 10.1136/bcr-2020-240613, PMID: 34404642 PMC8375734

[10] Facchinetti F Di Maio M Tiseo M . Adding PD-1/PD-L1 inhibitors to chemotherapy for the first-line treatment of extensive-stage small cell lung cancer: a meta-analysis of randomized trials. Cancers (Basel). (2020) 12:2645. doi: 10.3390/cancers12092645, PMID: 32947924 PMC7565587

[11] Kato M Uchida J . Recent advances in immune checkpoint inhibitors in the treatment of urothelial carcinoma: A review. Int J Urol. (2023) 30:1068–79. doi: 10.1111/iju.15278, PMID: 37602512

[12] Patel A Bisno DI Patel HV Ghodoussipour S Saraiya B Mayer T . Immune checkpoint inhibitors in the management of urothelial carcinoma. J Cancer Immunol. (2021) 3:115–36. doi: 10.33696/cancerimmunol.3.047, PMID: 34263255 PMC8276975

[13] Kato M Uchida J . Recent advances in immune checkpoint inhibitors in the treatment of urothelial carcinoma: A review. Int J Urol. (2023) 30:1068–79. doi: 10.1111/iju.15278, PMID: 37602512

